# Supplementary material for: Longitudinal associations between physical activity and other health behaviours during the COVID-19 pandemic: a fixed effects analysis
Source: Sci Rep. 2022 Sep 24;12:15956. doi: 10.1038/s41598-022-20196-6 (PMC9509399; doi:10.1038/s41598-022-20196-6)
Supplement: Supplementary file 1 — Supplementary Information. [file 41598_2022_20196_MOESM1_ESM.pdf]

Supplementary Material

Longitudinal associations between physical activity and other health behaviours during the COVID-19 pandemic: A fixed effects analysis.

Outcome (MET hours) derivation:

Metabolic equivalence of tasks (MET) hours used based on Ainsworth et al., 2011 [1]. Purposeful leisure time physical activity measured, and their decided MET equivalent encompassed:

i) Exercising inside your home or garden (e.g. doing yoga, weights or indoor exercise) (4 METs; taken as the mid-point of the stated activities)
ii) Going out for moderate or high intensity activity (e.g running, cycling or swimming) (6 METs; taken as the mid-point of the stated activities)
iii) Going out for a walk or other gentle physical activity (3.3 METs)

Ainsworth, B.E., et al., *2011 Compendium of Physical Activities: a second update of codes and MET values.* Med Sci Sports Exerc, 2011. **43**(8): p. 1575-81.

Supplementary table 1. Comparison of sample characteristics at baseline of smallest sample included in fixed effects models (nutrition quality outcome models) due to invariance in the outcome.

	Max excluded (Weighted) N=36,263	Min Included (Weighted) N=16,521
**Age (mean, SD)**	47.1 (17.3)	45.5 (16.7)
**Women**	50%	56%
**White Ethnicity**	87%	85%
**Highest level of education**		
No qualifications	7%	4%
Completed GCSE or equivalent (at school until age 16)	28%	24%
Completed post-16 vocational course	9%	7%
A-levels or equivalent (at school until age 18)	25%	26%
Undergraduate degree or professional qualification	19%	22%
Postgraduate degree	12%	16%
**What is your usual total household income per annum?**		
<£16,000	22%	12%
£16,000-£29,999	26%	22%
£30,000-£59,999	32%	37%
£60,000-89,999	12%	17%
£90,000-119,999	4%	7%
≥£120,000	4%	5%
**Time-varying sample characteristics at baseline**		
**Physical activity, MET hours (mean, SD)**	6.6 (9.7)	7.2 (9.6)
**Alcohol consumption**		
More than usual	27%	25%
**Sleep Quality**		
Good or Very good	31%	35%
**Diet Quantity**		
More than usual	32%	35%
**Nutrition Quality**		
More healthy	3%	3%
**Sedentary time, hours (mean, SD)**	8.1 (6.0)	7.9 (6.0)
**Financial adversity**	25%	26%
**Difficulty accessing essentials**	9%	7%
**PHQ-9 Score (mean, SD)**	8.2 (6.7)	8.0 (6.5)
**GAD-7 Score (mean, SD)**	6.0 (5.9)	6.0 (5.7)
**Self-isolation due to COVID exposure**	2%	2%
**Time-varying Effect Modifiers at baseline**		
**Clinically diagnosed mental health problem**	22%	22%
**Physical health condition**	41%	37%
**Overweight (BMI≥25)**	51%	56%

ST1. Comparison of excluded and included participants in fixed effects models of nutrition quality due to invariance in nutrition quality during participants waves contributed during the study period.

Supplementary table 2. Fixed effects regression models testing within-individual time-varying associations between physical activity, screen-based sedentary time, and creative sedentary time.

	**Independent Variable: Metabolic Equivalent of Task Hours During Last Weekday**					
	Fixed Effects Model additionally adjusting for time-varying confounders, other health behaviours.			***Fixed Effects Model additionally adjusting for time-varying confounders, other health behaviours and other sedentary activities.***		
**Health Behaviour**	**Coef.**	95% C.I.	*p*	**Coef.**	95% C.I.	*p*
Screen-based sedentary time	0.093	0.089-0.098	<0.001	0.076	0.072 - 0.080	<0.001
Creative sedentary time	0.039	0.036-0.041	<0.001	0.028	0.026 - 0.029	0.029

ST2. Sensitivity fixed effects models of PA and sedentary activities when subdivided into those considered more 'positive' or 'enriching' than other screen-based sedentary activities.
